# Serological Surveillance and Risk Factor Analysis for Parrot Bornavirus in Taiwan

**DOI:** 10.1155/2024/7811540

**Published:** 2024-04-13

**Authors:** Jing-Yuan Chen, Meng-Chi Wu, Zih-Syun Fang, Hui-Wen Chen

**Affiliations:** ^1^Department of Veterinary Medicine, School of Veterinary Medicine, National Taiwan University, Taipei, Taiwan; ^2^Animal Resource Center, National Taiwan University, No 1 Sec 4 Roosevelt Road, Taipei 10617, Taiwan

## Abstract

Parrots are traded globally and pose a substantial risk for disease transmission involving parrot-specific pathogens. Parrot bornavirus (PaBV) belongs to the *Bornaviridae* family and encompasses two clades: *alphapsittaciforme* (PaBV-1 to -4, PaBV-7, and -8) and *betapsittaciforme* (PaBV-5 and PaBV-6). These clades cause proventricular dilatation disease, a chronic disease affecting all parrot species. PaBV infections can persist for varying durations in parrots, but the transmission routes are still not well understood. Therefore, surveillance of PaBV-infected parrots is necessary for disease control and improving psittacine aviculture. This study used isolated PaBV-4 NTUCL7 and PaBV-5 NTUCL54 strains to establish and validate two serological diagnostic methods: immunoblotting (IB) and immunocytochemical staining (ICC). To determine the prevalence of PaBV in parrots in Taiwan, 370 clinical serum samples were collected from 13 collaborative veterinary hospitals during a 1-year surveillance period. Serological surveillance revealed a seropositivity rate of 25.68%. Among the seropositive samples, 91.58% were infected with *alphapsittaciforme* PaBV, demonstrating the predominance of this viral clade in parrots. An analysis of risk factors also demonstrated an association between seropositivity and parrot genera, age, and clinical signs. Cohen's kappa coefficient analysis showed a high degree of similarity (kappa value = 0.975) between the IB and ICC results, which shows that these serological diagnostic measures are robust. This study established two reliable serological diagnostic measures that are instrumental in serological surveillance, particularly in one of the major parrot-exporting regions. The surveillance results increase the understanding of PaBV infection and associated risk factors and allow methods to be devised for the conservation and protection of parrot populations.

## 1. Introduction

Parrots are among the most extensively traded companion animals globally. The main sources of parrot trade are regions of Central and South America, including Argentina, Peru, Uruguay, Guyana, Africa (Tanzania and Senegal), Asia (India, Indonesia, and Taiwan), and the Netherlands in Europe [1]. Widespread international trade in parrots amplifies the potential for disease transmission and increases the risk of psittacine aviculture in endangered species.

Parrot bornaviruses (PaBVs) belong to *orthobornaoviruses*, within the *Bornaviridae* family, and are the etiologies of proventricular dilatation (PD) disease in psittacine species [1]. In terms of the viral genome sequence, eight genotypes of PaBV have been classified into the *alphapsittaciforme* group (PaBV-1 to -4, PaBV-7, and -8) and *betapsittaciforme* group (PaBV-5 and -6). If a parrot is exposed to the virus, the virus can remain latent, and the parrot may present as asymptomatic for a variable period. When clinical signs develop, highly variable disorders, including mild-to-severe gastrointestinal symptoms and neurological disorders, are reported separately or simultaneously in clinical cases [2]. PaBV was discovered in 2008; however, more information on this fastidious viral disease is required, such as its diagnosis, epidemiology, transmission mechanisms, pathogenesis, and therapeutic or prophylactic measures [3]. Although PaBV infection has been reported mainly in parrots, some cases have revealed infection with avian bornavirus in nonpresumed primary hosts, such as a Himalayan monal (*Lophophorus impejanus*) naturally infected with PaBV-4 and the infection of avian-bornaviruses in eagles, gulls, and emu, suggesting the possibility of cross-species transmission [4–6].

For diagnosis, detection of the viral genome or presence of the virus is evidence of infection [2]. In terms of antigen detection, a previous study used recombinant proteins to generate virus-specific antibodies and developed a sandwich ELISA to detect the virus in tissue homogenates, cloacal swabs, and feather calami. Sandwich ELISA based on antibodies against phosphoproteins and matrix proteins is highly sensitive for virus detection, but the screening results for diagnosed parrots are variable [7]. Most previous studies used reverse transcription-polymerase chain reaction (RT-PCR) to detect viral RNA. Most RT-PCR assays detect a small fragment of a specific viral gene for a single strain of avian bornavirus [2, 3, 8–11]. Few RT-PCR assays involve a small range of closely related viruses [12, 13]. Previous studies using RT-PCR for PaBV surveillance revealed highly diverse prevalence rates between 2.1% and 54.1% in Asian countries, 28.6% in pet parrots, and 30.2% in free-ranging parrots in Brazil [14–19]. However, PaBV RNA can be detected intermittently, depending on the course of the infection, which significantly affects the detection results and allows the prevalence to be underestimated by RT-PCR [2].

Rather than detecting a viral nucleotide or viral antigen, serological diagnosis of a virus-specific antibody response provides direct evidence of a viral infection and an induced immune response. Previous studies have shown that a mammalian Borna disease virus (BoDV) infection in Madin–Daryby canine kidney cells (MDCK) was identified by PaBV-specific serum antibodies from infected parrots using an indirect immunofluorescence assay (IIFA) [20, 21]. However, the cross-reaction of serum antibodies to BoDV may not cover the eight genotypes of PaBV, and the diversity of antigenicity between the BoDV and PaBV structural proteins may affect the detection results [22]. Therefore, IIFA was only used to determine the antibody response in experimentally infected parrots [8, 9]. Some studies have reported that serum antibodies can also be used to identify homogenates of PaBV-infected duck embryo fibroblast cells or recombinant viral nucleocapsid protein and phosphoprotein using an immunoblotting (IB) assay [22, 23]. However, there has been no validation or serological surveillance using IIFA or IB; hence, more studies using reliable serological diagnostic measures and surveillance for PaBV in parrots are necessary. This study is the first to establish and validate the use of PaBV virions in IB and virus-infected monolayer cells in immunocytochemical staining (ICC) for serological diagnosis and serum surveillance of parrots. Surveillance results from high specificity and sensitivity IB and ICC updated the serologic prevalence of PaBV in parrots in Taiwan, and the analysis of related risk factors is a valuable clinical diagnostic reference for veterinarians.

## 2. Materials and Methods

### 2.1. Viruses and Cells

Two different PaBV genotypes were identified in naturally infected parrots in Taiwan, which were subsequently isolated using serial passaging in QM7 cells. Next-generation sequencing analysis was performed for the isolated virus strains, and the complete genome was submitted to the GenBank database as the PaBV-4 NTUCL7 strain (GenBank: OM939725) and PaBV-5 NTUCL54 strain (GenBank: OM777141).

To propagate the virus, QM7 cells were persistently infected with PaBV-4 or PaBV-5, maintained in M199 medium (11150059, Thermo Fisher™) supplemented with 10% FBS and 1% Antibiotic–Antimycotic (15240062, Thermo Fisher™), and incubated at 37°C in a 5% CO_2_ atmosphere. When the virus stock was produced, the cells were subjected to repeated freeze–thaw cycles and concentrated using an Amicon® filter centrifuge (UFC901024, Merck). After concentration, Bradford assay was performed to determine the amount of protein in the concentrated virus. Concentrated PaBV-4 NTUCL7 and PaBV-5 NTUCL54 strains were used to establish serological diagnostic criteria. Additionally, a 50% tissue culture infectious dose (TCID_50_) assay was conducted to determine the infectious virus titer of the concentrated virus. Briefly, QM7 cells were seeded onto 96-well plates 1 day before the experiments, and the virus was incubated with the cells for 24 hr. The virus was then removed, and the cells were washed. After 7 days, the cells were fixed with 80% acetone and stained with mouse anti-PaBV nucleoprotein serum. The titer was calculated using the Reed–Muench method [24].

### 2.2. Serum Samples

To determine the seroprevalence of PaBV in Taiwanese parrots, a collaborative surveillance program involving veterinary hospitals was conducted between 2021 and 2022. These samples were submitted by the participating hospitals as representative samples to determine the seroprevalence of PaBV in parrots in Taiwan. Clinical signs were recorded and reported by the clinical veterinarians who participated in the program, including respiratory signs (cloudy air sac, ocular and nasal discharge, dyspnea, cough), gastrointestinal signs (delayed passage of the ingesta, shedding of undigested seeds and diarrhea, weight loss, anorexia, and PD), and neurological disorders (feather plucking, self-mutilation, incoordination, seizures, tremors, lameness, depression, and blindness) to analyze risk factors.

The serodiagnosis results of a small-scale pilot study were used to calculate the minimum sample size for surveillance to produce a statistically representative result. For preliminary small-scale screening, the estimated prevalence of PaBV was approximately 40%; therefore, ≥369 serum samples were required for a 95% confidence level. The actual value was within ±5% of the estimated value [25].

### 2.3. IB Assay

Concentrated PaBV-4 and PaBV-5 virions were used for IB assay using the method of previous studies with some modifications [22, 23]. One microgram of PaBV-4 or PaBV-5 virions treated with a sample buffer was electrophoresed on 10% SDS-PAGE and transferred to a nitrocellulose membrane (NBA085C001EA, PerkinElmer). The membranes were blocked with 5% skim milk (23,210, BD) and then incubated with 1 : 100 diluted parrot serum at room temperature for 1 hr. The membrane was washed with PBST and incubated with anti-bird IgG conjugated with HRP (A140-110P, Bethyl) for 1 hr. After repeated washes, the protein signal was developed using Clarity™ Western ECL substrate (170-5061, Bio-Rad) and visualized using the ChemiDoc XRS^+^ System with Image Lab Software (version 5.2.1, Bio-Rad).

### 2.4. ICC Assay

PaBV-4 is the predominant strain in parrots; therefore, the PaBV-4 NTUCL7 strain was used for an ICC assay using a previously described method, with some modifications [26–28]. Briefly, QM7 cells were seeded in 96 flat-well plates and infected with PaBV-4 NTUCL7 strain at a multiplicity of infection of 0.05. The infected cells were then fixed, permeabilized using frozen acetone, and blocked using 5% normal goat serum (005-000-121, Jackson ImmunoResearch Laboratories) as an antigen plate for diagnosis. Parrot serum was diluted with 5% normal goat serum at 1 : 100 or fourfold serially diluted (1 : 40 to 1 : 2,560) and incubated with the antigen plate. Serum IgG was further recognized using HRP-conjugated anti-bird IgG, and the DAB^+^ substrate chromogen system (K3468, Dako) was used for color development. The dark brown signal that appears in the cytoplasm of the infected cells represents a positive response. When the serum sample showed a PaBV-specific antibody response for serologic diagnosis, RT-PCR and nucleotide sequencing specific to the M gene were performed to determine the viral genotype [29].

### 2.5. Evaluation of Risk Factors and Statistical Analysis

The odds ratio (OR) for risk factors, including breed, sex, age (older age: older than 1 year old; juvenile: younger than 1 year), genus, household of multiple or single birds, and clinical signs, was calculated using Fisher's exact test, and a value for *p* < 0.05 means that the result was statistically significant. To determine whether the detection results for these two serological measures were in agreement, IB and ICC were compared using 40 seropositive and 40 seronegative samples. McNemar's test and Cohen's kappa coefficient analysis were performed to determine how the two serological diagnostic measures are correlated for this study. Data were analyzed using GraphPad Prism, Version 9.4.1 (GraphPad Software, San Diego, California, USA) and the R program (Version 4.2.3) [30].

## 3. Results

### 3.1. Validation of Serologic Diagnostic Measures

**Figure 1:**
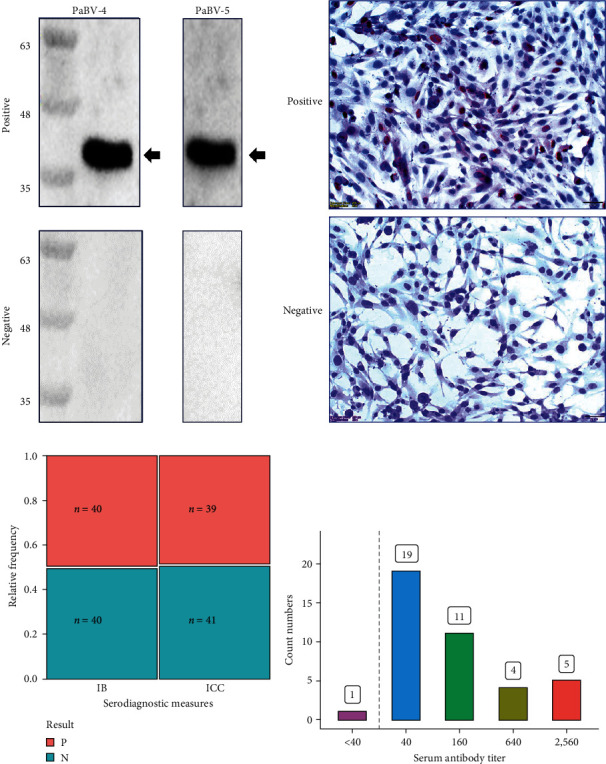
Validation of serological diagnostic measures for this study. (a) One microgram of sample buffer-treated PaBV-4 or PaBV-5 virions was electrophoresed and transferred to a nitrocellulose membrane for the IB assay. After blocking with skim milk, the membrane was incubated with positive or negative parrot serum at 1 : 100 dilution. (b) For the ICC assay, PaBV-4 NTUCL7 strain-infected monolayer QM7 cells were incubated with positive or negative parrot serum to identify PaBV-infected QM7 cells. (c) To verify the consistency of IB and ICC detection results, 40 seropositive and 40 seronegative samples were used to validate the two serological diagnostic measures. (d) The bar chart demonstrates the antibody titer distribution for PaBV-positive serum samples. Each bar indicates the number of positive samples for each titer.

In this study, IB and ICC were established to analyze PaBV-specific antibodies in the serum samples. The results showed a robust positive signal for the viral nucleocapsid protein, a highly conserved immunodominant structural protein for *alphapsittaciforme* PaBVs, at approximately 40 kDa in IB (Figure 1(a)) [13, 31]. In ICC, the serum antibody recognized PaBV-4-infected QM7 cells, producing a brown coloration in the cytoplasm (Figure 1(b)). To evaluate the coordination of the serological diagnostic methods, a total of 80 serum samples were randomly selected from the 370 samples that were analyzed, with 40 of the samples testing positive for IB and 40 testing negative. These samples were then subjected to analysis using ICC to determine the antibody response and titration. In comparison with IB, which differentiated all positive and negative serum samples, ICC identified 39 of the 40 seropositive samples and all 40 seronegative samples (Figure 1(c)). McNemar's test (*p* < 0.05) shows that there is significant agreement between the results for IB and ICC. Cohen's kappa coefficient gives a kappa value of 0.975, with a 95% confidence interval of 0.93–1.02; therefore, there is a high degree of agreement between the two detection methods. The 40 positive serum samples were serially diluted fourfold for the ICC assay, and antibody titers were determined. The results are shown in Figure 1(d). Only one seropositive sample demonstrated a weak antibody response (titer < 1 : 40). Most serum samples had an antibody titer of 1 : 40 (*n* = 19; 47.5%). Considering the high sensitivity of the IB method, it was subsequently employed for clinical serum antibody screening.

### 3.2. Serum Samples for the Study

**Figure 2:**
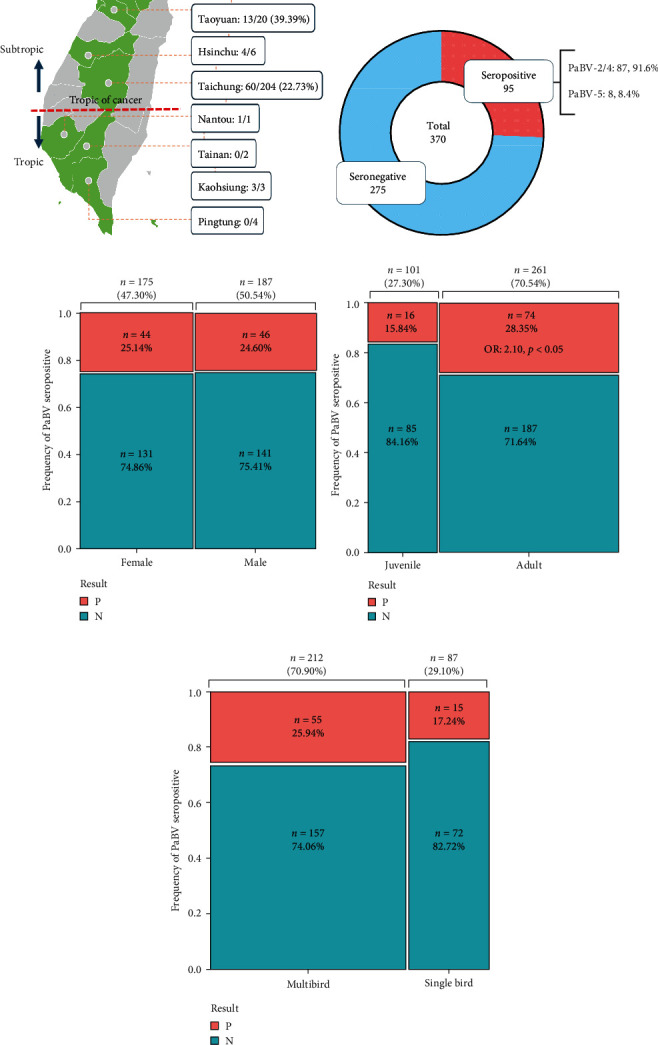
Summary of serum samples used in this study. (a) Clinical serum samples collected from various cities in Taiwan. Data for seropositive, seronegative, and seropositive rates are presented. (b) The frequencies of seropositive (*n* = 95) and seronegative (*n* = 275) samples and the identified PaBV genotypes (PaBV-2/4, *n* = 87, and PaBV-5, *n* = 8) are shown. (c) The frequency of serum samples (*n* = 370) from male (*n* = 187) and female (*n* = 175) parrots was analyzed to determine the effect of sex. Seven samples without sex information were excluded from Fisher's exact test. (d) The frequency of serum samples from juvenile (<one year old, *n* = 101) and adult (>one year old, *n* = 261) parrots was evaluated using Fisher's exact test. (e) The frequency of serum samples from multibird and single-bird households was assessed using Fisher's exact test. The odds ratio (OR) was calculated and statistical significance was determined using a *p*-value of less than 0.05.

Parrot serum samples were collected from 13 veterinary hospitals in eight cities in Taiwan. Taipei, Taoyuan, Hsinchu, Nantou, Taichung, Tainan, Kaohsiung, and Pingtung. A total of 370 serum samples from 18 different parrot genera were included in this study (Figure 2(a)). Of the 370 serum samples, 95 were seropositive (25.68%), and 275 were seronegative (74.32%). Among the seropositive samples, 91.58% (87/95) showed a specific antibody response to PaBV-4, and 8.42% (8/95) showed a specific PaBV-5 antibody response, indicating that this was the dominant *alphapsittaciforme* PaBV in Taiwan (Figure 2(b)). Furthermore, among all the serum samples submitted, 187 were collected from male parrots (50.54%), 175 were collected from female parrots (47.30%), and seven samples were not assigned sex information (Figure 2(c)). A total of 101 samples were collected from juveniles (<1 year old), 261 samples were collected from adults (>1 year old), and eight samples had no age information (Figure 2(d)). Serum samples were collected each month of the year. The results revealed a lower seropositive rate in May and higher seropositive rates in October and November (*Supplementary 1*).

### 3.3. PaBV Is Specific to Certain Genera of Parrots

The frequency of parrot genera and surveillance results are shown in Table 1. Seropositive results were observed in 15 of the 18 genera. *Psittacus* (*n* = 91, 24.59%) was the most commonly submitted genus, followed by *Cacatua* (*n* = 69, 18.65%), *Ara* (*n* = 54, 14.59%), *Eclectus* (*n* = 40, 10.81%), and *Pionites* (*n* = 34, 9.19%). These are the most commonly encountered parrots in Taiwan. For these common genera, the positivity rates for *Psittacus*, *Cacatua*, *Ara*, *Eclectus*, and *Pionites* were 27.47%, 37.68%, 22.22%, 12.50%, and 17.65%, respectively.

Fisher's exact test was used to determine whether PaBV seropositivity was observed in a specific genus for which more than three samples were submitted. Of the 12 genera, *Cacatua* had a high OR of 2.03 (*p* < 0.05); therefore, it is highly likely to be susceptible to PaBV infection in Taiwan, in agreement with previous studies reported in Japan and Germany [16, 32]. The results for the other commonly encountered parrots, *Aratinga* (*n* = 9, OR: 2.37), *Guaruba* (*n* = 8, OR: 2.97), *Pyrrhura* (*n* = 3, OR: 5.86), and *Psittacus* (*n* = 91, OR: 1.13), feature a *p*-value greater than 0.05 for Fisher's exact analysis and a higher OR than other genera. Apart from the genera with a high OR, *Amazona* (OR: 0.21), *Ara* (OR: 0.8), *Myiopsitta* (OR: 0.84), *Pionites* (OR: 0.60), *Nymphicus* (OR: 0.72), *Poicephalus* (OR: 0.28), and *Eclectus* (OR: 0.38) had an OR of less than 1; therefore, these genera may be less susceptible to PaBV (Table 1). The dynamics of OR may depend on the survey population; therefore, further surveillance may provide a more accurate estimate of genus factors. This study is the first risk analysis of different genera from serosurveillance results.

### 3.4. Age Is a Risk Factor for PaBV Seropositive

The number of samples of different genera that were submitted depended on ubiquity; however, there were similar numbers of male (*n* = 187, 50.54%) and female (*n* = 175, 47.57%) parrots. The results showed that there were no statistical differences in the PaBV seropositive rates between male parrots (46/187, 24.60%) and females (44/175, 25.14%) (Figure 2(c)).

Life span varies significantly depending on the parrot genera, but most parrots can be weaned and ingested independently at 1 year of age; therefore, this study determined the OR for the seropositive rate in serum samples for birds that were older than 1 year (elder, *n* = 261) or less than 1 year (juvenile, *n* = 101). In total, 74 serum samples (28.35%) from the elderly parrot population were seropositive, whereas only 16 juvenile samples (15.84%) were seropositive. Fisher's exact test showed that the OR was 2.10, and the *p*-value is 0.01 for the age factor, so the older population was more likely to be seropositive for PaBV (Figure 2(d)).

In addition to age and sex, this study also determined whether the number of birds in a household was correlated with a seropositive result (Figure 2(e)). Of the 370 serum samples used in this study, 299 contained information on household composition. Of the 299 serum samples, 212 (70.90%) were from households with multiple birds, and 87 (29.10%) were from single-bird households. Fisher's exact test shows that the OR is 1.68, and the *p*-value is 0.13 for multibird households. These results indicate that the presence of multiple birds in a household does not significantly increase the risk of a PaBV seropositive result.

### 3.5. Clinical Signs Associated with PaBV Infection

**Figure 3:**
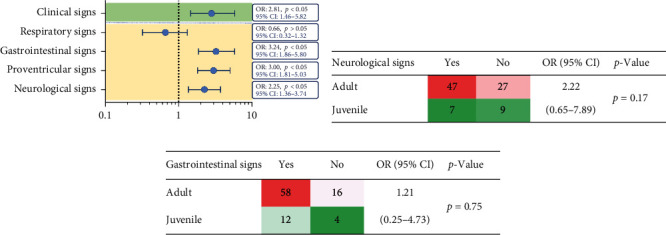
Correlation between clinical signs and serodiagnostic findings. (a) Various clinical signs, including respiratory, gastrointestinal and neurological signs, were examined as risk factors for PaBV seropositive results. To evaluate these associations, Fisher‘s exact test was employed to calculate the odds ratio (OR) and the corresponding 95% confidence interval (95% CI). The results are presented as OR ± 95% CI. The correlations between age and observed neurological signs (b) and gastrointestinal signs (c) were summarized and analyzed with Fisher's exact test.

To determine whether there was a correlation between PaBV serologic diagnostic results and the presence of clinical signs, such as respiratory, gastrointestinal, and neurologic signs, were studied as potential risk factors (Figure 3(a)). Among the samples used in this study, 272 (73.51%) showed at least one type of clinical sign, and 82 (30.15%) were seropositive for the PaBV antibody, with an OR of 2.81 (*p* < 0.05), and clinical signs were correlated with the serodiagnostic result (*Supplementary 2*). In the seropositive population, respiratory symptoms (OR: 0.66, *p* > 0.05) were not associated with PaBV; however, parrots that exhibited gastrointestinal symptoms (OR: 3.24, *p* < 0.05) or neurological disorders (OR: 2.25, *p* < 0.05) were likely to be seropositive for PaBV. Proventricular dilation was also significantly associated with seropositivity result (OR: 3.00; *p* < 0.05) (Figure 3(a)). As age is a crucial factor associated with seropositive results, we further evaluated the correlation between neurological and gastrointestinal signs and age. Although a high OR was observed, the results did not show a statistically significant correlation between these factors (Figures 3(b) and 3(c)). Overall, these results demonstrate that clinical signs can indicate PaBV infection and are valid indicators for clinical diagnosis.

### 3.6. Paired PaBV Seropositive Samples

Infected birds do not consistently spread the virus or develop a specific antibody response; therefore, paired sera from naturally infected parrots provide information regarding PaBV infection [2]. The history, clinical signs, and diagnostic results of the four serum samples: 1 for PaBV-2, 2 for PaBV-4, and 1 for PaBV-5 are listed in Table 2. The four PaBV-infected parrots developed PD and showed clinical signs, and the parrot infected with PaBV-2 developed PD within 1 month of a seropositive result. There is a long-lasting antibody response in *Cacatua* infected with PaBV-4, which may persist for 9 months. The PaBV4-specific antibody response, PD, and perceptible PaBV-4 RNA level were also detected within 2 months in an infected *Psittacus*. *Eclectus* infected with PaBV-5 demonstrated feather plucking and PD at the beginning of the disease course. There was also an intermittent viral RNA detection result and a dynamic antibody response after 4 months.

## 4. Discussion

Parrots are among the most widely traded wildlife species. It is sold as a companion and captive animal in human society because it is clever and features beautiful plumage [33–35]. In addition, PaBV infection occurs worldwide in captive parrots and is one of the most fastidious pathogens for zoos and the rehabilitation of endangered species [2]. This study used serological diagnostic measures to profile the prevalence of PaBV infection in parrots in Taiwan, determine associated risk factors, and calculate the risk of contracting PaBV.

Some previous studies have provided information on the positive rate of PaBV in Japan, China, Thailand, Malaysia, and Brazil; however, the restricted sample size and limitations of RT-PCR can underestimate the prevalence of PaBV [14–19]. The population for this study consisted of 370 serum samples from different cities in Taiwan, which satisfied the epidemiological and statistical analysis requirements for PaBV surveillance and minimized bias for a limited sample size compared with previously reported results. This study showed a seropositive rate of 25.68%, and a specific antibody response for *alphapsittaciforme* PaBV (PaBV-2 or PaBV-4, 91.6%) was predominant in Taiwan. In Thailand in 2019, a similar predominant frequency of *alphapsittaciforme* PaBV was reported (96% for PaBV-2 and PaBV-4, and 4% for PaBV-5), reflecting the vibrant trade between Taiwan and Southeast Asian countries; therefore, PaBV surveillance is necessary to mitigate transboundary disease transmission [14].

Furthermore, the surveillance results of this study also showed that serum samples from adult parrots are more likely to be seropositive, corresponding to the results of a recently published survey of pet parrots in Malaysia [15]. Moreover, previous studies have described different seroconversion, pathological changes, and viral tissue distribution in an experimental PaBV-4 inoculation experiment in juvenile parrots (age at inoculation: 1–6 days) and adult parrots (age at vaccination: 1–5 years). These studies profiled an age-dependent manner in the PaBV-induced inflammatory response [36, 37]. However, no association was observed between the frequency of clinical symptoms and age in our serological surveillance study, suggesting a gap between the experimental animal model and clinical observations. Additionally, another study indicated that no contact infections were observed in canaries and cockatiels experimentally infected with avian-bornavirus [38]. This study collected more than 70% of serum samples from households with multiple birds, but the analysis results showed that having numerous birds in a family did not significantly increase the risk of positive PaBV serology. To the best of our knowledge, this study is the first attempt to correlate experimental results with naturally infected clinical findings, revealing fundamental conflicts. Further research and appropriate animal trials are required to explore these conflicts, providing advanced information to improve the treatment of infected parrots and the development of vaccines or drugs.

Persistent infection is a characteristic of PaBV infection, and different genotypes of PaBV can produce various disease outcomes during prolonged infection [39]. Among *alphapsittaciforme* viruses, experimentally, PaBV-2-infected parrots experience more severe clinical signs and disease progression than birds that are experimentally infected with PaBV-4. Furthermore, PaBV-2 infection was reported to mainly affect the gastrointestinal system, whereas PaBV-4 infection produced more neurological symptoms in experimental infection trials [10, 20]. A similar situation has been observed in naturally infected parrots. Clinical samples with paired serum for this study recorded a PaBV-2-infected parrot that was reported to have digestive problems on the first visit to the clinic, whereas the two PaBV-4-infected parrots visited the clinics due to neurological symptoms. Additionally, an analysis of the paired serum of a PaBV-5-infected parrot is summarized in this study. Compared with PaBV-4 infection, which may induce a prolonged antibody response for 9 months, the antibody response for PaBV-5 may develop and decrease rapidly within 2 months. Since PaBV-5 infection is rarely reported in clinical cases, this is the first investigation to provide a comprehensive paired serum screening result for a naturally PaBV-5-infected parrot [2]. By analyzing paired serum samples and comparing the serologic diagnostic results and clinical outcomes in PaBV-2-, PaBV-4-, and PaBV-5-infected parrots, our research provides valuable information for advanced research and clinical applications.

Titration of the antibody levels from the serum samples revealed that PaBV infection may induce a mild antibody response in most clinical cases. Previous studies using BoDV-infected MDCK cells to generate antigen plates and IIFA to determine antibody titer levels in parrot serum showed antibody titers of 1 : 160 and 1 : 20,480 [21, 39]. Although some immunological cross-reactions between BoDV and PaBV have been evaluated, it is not yet known whether the coordination of antibody responses for the two viruses has been accomplished. Therefore, the antibody response induced by PaBV infection has not yet been clarified or evaluated. This study validates the high agreement between two PaBV-based serological diagnostic measures and accordingly determines the distribution of the antibody titer from 40 seropositive samples, which may provide more specific information on the natural PaBV infection-induced antibody response in parrots and improve veterinarian's knowledge in clinical diagnosis.

## 5. Conclusions

This study established two serological diagnostic measures and determined the serological prevalence of PaBV infection in parrots in Taiwan. Risk factor analysis showed that several factors were associated with the PaBV seropositivity rate, including the genus *Cacatua*, adult age, and clinical signs. These findings provide valuable information that can help refine quarantine strategies for disease prevention and guide future animal trials to elucidate the pathogenesis of PaBV infections. Furthermore, a comprehensive summary of clinically paired serum information and an analysis of clinical signs allows a better understanding of the disease risk factors and clinical manifestations and allows veterinarians to provide an accurate clinical diagnosis of PaBV infection and more effective care and treatment for infected birds. In general, this study contributes to the knowledge of PaBV infection and offers crucial information to protect parrot populations.

## Figures and Tables

**Table 1:** Summary of detected results for submitted parrot genera.

Genera	Seropositive/total cases	Positive rate (%)	Odds ratio (OR)	*p*-Value
*Amazona*	1/14	7.14	0.21	>0.05
*Ara*	12/54	22.22	0.80	>0.05
*Aratinga*	4/9	44.44	2.37	>0.05
*Guaruba*	4/8	50.00	2.97	>0.05
*Myiopsitta*	5/22	22.73	0.84	>0.05
*Pionus*	1/1	—	—	—
*Pionites*	6/34	17.65	0.60	>0.05
*Pyrrhura*	2/3	66.67	5.86	>0.05
*Neopsephotus*	1/1	—	—	—
*Cacatua*	26/69	37.68	2.03	0.01
*Nymphicus*	1/5	20.00	0.72	>0.05
*Poicephalus*	1/11	9.09	0.28	>0.05
*Psittacus*	25/91	27.47	1.13	>0.05
*Agapornis*	1/2	50.00	—	—
*Eclectus*	5/40	12.50	0.38	0.054
*Psittacula*	0/2	0.00	—	—
*Melopsittacus*	0/1	—	—	—
*Trichoglossus*	0/3	0.00	—	—

The positive rate is shown for the genera with seropositive results for which more than one sample was submitted. Fisher's exact test was also performed to calculate the odds ratio for genera for which more than three serum samples were submitted. Differences were considered statistically significant if the *p*-value was less than 0.05.

**Table 2:** Parrots with paired serum samples used in this study.

PaBV genotypes	Genus of birds	Age	Clinic visit	Clinical symptoms	Observation of PD	PaBV-specific antibody^a^	PaBV antigen^b^
PaBV-2	*Cacatua* *(Cacatua moluccensis)*	1 year	1st time	Weight loss, anorexia, regurgitation	NA ^*∗*^	Positive	NA
			2nd time (1-month interval)	Weight loss, anorexia, proventricular dilatation	Positive by X-rays	Positive	Positive

PaBV-4	*Cacatua* *(Cacatua moluccensis)*	2 years to 3 years 8 months	1st time	Posture problem	NA	Positive	NA
			2nd time (9-month interval)	Lameness, weight loss, anorexia, diarrhea, vomiting	NA	Positive	Positive

PaBV-4	*Psittacus* *(Psittacus erithacus)*	2 months to 4 months	1st time	Depression	NA	Negative	NA
			2nd time (2-month interval)	Depression, feather plucking, weight loss, undigested seeds in feces, proventricular dilatation	Positive by X-ray and palpation	Positive	Positive

PaBV-5	*Eclectus* *(Eclectus roratus)*	1 year	1st time	Proventricular dilatation Head shaking, wing shivering	Positive by X-ray	Negative	Positive
			2nd time	Proventricular dilatation, Feather plucking	Positive by X-ray	Positive	Negative
			3rd time	Proventricular dilatation, Feather plucking	Positive by X-ray	Positive	Negative
			4th time (1-month interval)	Proventricular dilatation, anorexia Feather plucking	Positive by X-ray	Negative	Negative

^*∗*^NA: not analyzed; ^a^Antibody was analyzed by immunoblotting assay; ^b^Viral antigen was detected by RT-PCR and sequencing specific for M gene. Four serum samples, one for PaBV-2, two for PaBV-4, and one for PaBV-5, with a detailed history, clinical symptoms, and diagnostic results, are listed.

## Data Availability

All data used and analyzed in this study can be obtained from the corresponding author upon reasonable request.

## Supplementary Materials

Supplementary 1 Summary of serum samples submitted each month.

Supplementary 2 Summary of clinical signs and serodiagnostic results.
