# Supplementary material for: Serological Surveillance and Risk Factor Analysis for Parrot Bornavirus in Taiwan
Source: Transbound Emerg Dis. 2024 Apr 13;2024:7811540. doi: 10.1155/2024/7811540 (PMC12017039; doi:10.1155/2024/7811540)
Supplement: Supplementary 1 — Summary of serum samples submitted each month. [file 7811540.f1.docx]

**Supplementary Table 1.** **Summary of serum samples submitted at each month.** The submitted samples, the seropositive samples, and the positive rates for each month were summarized.

| **Month** | **Submitted samples** | **Seropositive samples** | **Positive rate** |
| --- | --- | --- | --- |
| January | 24 | 8 | 33.33% |
| February | 16 | 2 | 12.50% |
| March | 40 | 7 | 17.50% |
| April | 32 | 7 | 21.88% |
| May | 36 | 3 | 8.33% |
| June | 28 | 9 | 32.14% |
| July | 25 | 3 | 12.00% |
| August | 29 | 7 | 24.14% |
| September | 28 | 5 | 17.86% |
| October | 41 | 18 | 43.90% |
| November | 39 | 17 | 43.59% |
| December | 31 | 9 | 29.03% |
