# Supplementary material for: Serological Surveillance and Risk Factor Analysis for Parrot Bornavirus in Taiwan
Source: Transbound Emerg Dis. 2024 Apr 13;2024:7811540. doi: 10.1155/2024/7811540 (PMC12017039; doi:10.1155/2024/7811540)
Supplement: Supplementary 2 — Summary of clinical signs and serodiagnostic results. [file 7811540.f2.docx]

**Supplementary Table 2. Summary of clinical signs and serodiagnostic results.** The number of serodiagnostic results and clinical signs were summarized.

| **Clinical signs** | | **Seropositive** | **Seronegative** |
| --- | --- | --- | --- |
| At least one clinical sign recorded | Yes | 82 | 190 |
|  | No | 13 | 85 |
| Respiratory signs | Yes | 13 | 82 |
|  | No | 53 | 222 |
| Gastrointestinal signs | Yes | 73 | 22 |
|  | No | 139 | 136 |
| Proventricular signs | Yes | 60 | 35 |
|  | No | 98 | 177 |
| Neurological signs | Yes | 56 | 39 |
|  | No | 107 | 168 |
